# The Curing Rheokinetics of Epoxyphosphazene Binders

**DOI:** 10.3390/ma13245685

**Published:** 2020-12-12

**Authors:** Natalia V. Bornosuz, Irina Yu. Gorbunova, Vyacheslav V. Kireev, Denis V. Onuchin, Mikhail L. Kerber, Viktoria V. Petrakova, Ivan A. Kryuchkov, Roman E. Nevskiy, Alexey V. Sokovishin, Venera V. Khammatova, Igor S. Sirotin

**Affiliations:** 1 Faculty of Petrochemistry and Polymer Materials, Mendeleev University of Chemical Technology, 125047 Moscow, Russia; bornosuz@muctr.ru (N.V.B.); igorbunova@muctr.ru (I.Y.G.); kireev@muctr.ru (V.V.K.); donuchin@muctr.ru (D.V.O.); kerber.k@yandex.ru (M.L.K.); vvvorobeva@muctr.ru (V.V.P.); 2 Division of Materials Science, Dukhov Automatics Research Institute (VNIIA), 125047 Moscow, Russia; ivan_kr@mail.ru (I.A.K.); mailbox75@vniia.ru (R.E.N.); vniia@vniia.ru (A.V.S.); 3 Design Department, Kazan National Research Technological University, 420111 Tatarstan, Russia; venerabb@mail.ru

**Keywords:** epoxy resins, diaminodiphenylsulfone, epoxyphosphazenes, rheokinetics

## Abstract

The influence of epoxyphosphazene-modifying additives on the features of the hot curing process of epoxy-amine composition was studied by the rotational viscometry method. The modification caused an acceleration of the curing process, changed rheokinetics of viscosity increase, especially the stage molecular mass growth of linear chains became almost twice shorter for composition with 30% modifier than for unmodified one. We suggest the reason for these changes is the polyfunctionality of epoxyphosphazene, which finally results in high-density network formation. In cold curing process the bulkiness of epoxyphosphazene molecule and the lack of heat for its motion results in incomplete cure. Thus, in order to cope with these difficulties hot curing systems were proposed and studied.

## 1. Introduction

Epoxy resins and epoxy-amine binders are widely used in various fields of technology due to their high adhesion to various substrates, mechanical properties, chemical resistance, the ability to cure with low shrinkage without the emission of low molecular weight by-products. Performance characteristics, in combination with the economic benefits of epoxy polymers, make them superior to many other classes of thermosetting polymers [1].

Modern industry requires a variety of epoxy-amine binders. In high-demand applications, high-temperature curing epoxy-amine systems are commonly used. A significant problem with unmodified epoxy binders in the cured state is low toughness, insufficient strength, rigidity, heat resistance, and high flammability [1].

One way to eliminate these drawbacks is the modification of epoxy binders, in particular with additives of compatible epoxides with higher functionality, which molecules are incorporated into the polymer network formed during curing [1,2].

A new promising class of modifiers in this field are polyfunctional epoxyphosphazenes, which have proved to be effective halogen-free flame retardants [3,4,5,6,7]. They are well compatible with epoxy matrix and can also improve mechanical properties [8], probably due to the formation of a special three-dimensional polymer network, in the nodes of which phosphazene cycles are located [3]. It is also known, however, that functional epoxyphosphazenes are solids or highly viscous liquids [9], which can hinder their processing [10]. Thus, for the successful use of epoxyphosphazenes as a component of epoxy binders, it is necessary to study in detail rheological behavior during the curing of compositions with different concentrations of the modifier.

This study would allow one to comprehend the influence of epoxyphosphazene on the viscosity increase during curing of epoxy-amine binders, whether compositions with modifiers are processable and suppose what processing method they fit more. However, we must note that this study concerned only the part of all technological properties that are necessary to define before choosing the processing method; in particular, we focused on the process of curing till gelation.

Currently, in many works, the process of curing is studied by differential scanning calorimetry (DSC) [11,12,13,14]. DSC method is universal and allows to create quite accurate models of curing, which further can be utilized either for curing regime predictions or for warpage calculations, for example. In this field of DSC studies, there are a lot of recent works dedicated to studies of accelerators and catalysts’ influence on the curing kinetics [14,15,16]. In Vyazovkin works, great attention is devoted to the evaluation of activation energy during the process [12,17,18]. However, in the case of gelation, DSC does not always represent unambiguously the processes leading to the curing of the oligomer matrix. Some unique works combine different monitoring methods to conduct a comprehensive view of the process of curing. For example, in Gorbunova, I.Yu and Malkin, A.Ya.’s study [19], the author’s goal was to investigate the kinetics of curing by the two methods being compared: rheology, which makes it possible to substantiate the technological recommendations for the selection of the curing regime, and traditional calorimetry, which represents the kinetics of elementary reactions occurring during the curing. Various aspects of the rheology of curing processes, including Т-Т-Т diagrams (time-temperature-transformation) [20], homogeneous and heterogeneous curing, the possibility of relaxation transitions in curing, were examined in the survey [21]. The study of the time-temperature regime of curing determines both the technological parameters of the process and the properties of the final products.

This work is devoted to the study of the isothermal process of curing of general bisphenol-A-based epoxy resin modified by epoxyphosphazene additives with diaminodiphenylsulfone curing agent till gel point by rotational viscometry. In particular, the influence of the modifier concentration and temperature on the main rheological parameters of the curing was revealed.

## 2. Materials and Methods

The formulations based on general bisphenol-A-epoxy resin, epoxyphosphazene modifier with diaminodiphenylsulfone curing agent were studied.

Bifunctional bisphenol-A-based epoxy resin of ED-20 brand (satisfied the Russian standard GOST 10587-84), manufactured at the Y.M. Sverdlov plant (Dzerzhinsk, Russia), Mn = 390 g/mol, epoxy group content of 20.0–22.0%, and dynamic viscosity of 18.4 Pa·s at 25 °C, was used as the epoxy resin matrix.

**Figure 1:**
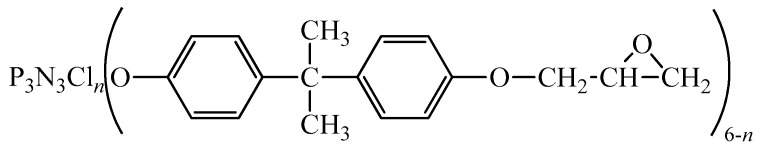
The general formula of the epoxyphosphazene oligomer (EP), where n = 0–2.

An epoxyphosphazene resin, which is a homogeneous mixture of a conventional bisphenol-A-based epoxy oligomer and epoxyphosphazene oligomer (EP) of the following general formula (Figure 1), was obtained in Mendele ev University of Chemical Technology (Russia) according to the technique [22] in such a way that EP content in epoxyphosphazene resin was 50% by weight. Epoxy group content in epoxyphosphazene resin was 17-19%, chlorine content was 2.5%.

Epoxyphosphazene resin was added to ED-20 to obtain EP content of 0%, 10%, 20%, 30% by weight in the resulting matrix (Table 1).

As the curing agent, diaminodiphenylsulfone (DDS) of the Aradur 9664-1 brand from Huntsman (Germany) was used in the form of a fine powder with a particle size of less than 64 μm and a Tm = 175 °C. It was added to the above-mentioned mixtures in a stoichiometric ratio.

The calculated amount of ED-20 and epoxyphosphazene resin was mixed on a stirrer at 80 °C for 10 min to achieve a homogeneous mixture. The calculated amount of DDS was added to the matrix and stirred for 20 min at 125 °C till complete dissolution. Subsequent degassing of the system was performed at 125 °C for 15 min at a residual pressure of 1.0 kPa. At the end of the degassing process, the resulting compositions were used as received for further curing study.

The viscosity of the formulations during their curing process prior to the gel point was studied. The measurements were carried out on a modular rheometer Physica MCR 302 (Anton Paar, Austria) with a cone-plane working node (angular = 1°). The following conditions were provided for measurements: angular velocity 1.05 rad/s, gap 0.5 mm, prerotation during 2 min. We conducted experiments at isotherms 160 °C, 170 °C, and 180 °C. For each formulation, at least 3 tests were carried out to achieve the convergent results.

## 3. Results and Discussions

For the rheokinetic studies, the above-mentioned four systems were chosen, which made it possible to evaluate the contribution of the modifier to the features of the curing process.

**Figure 2:**
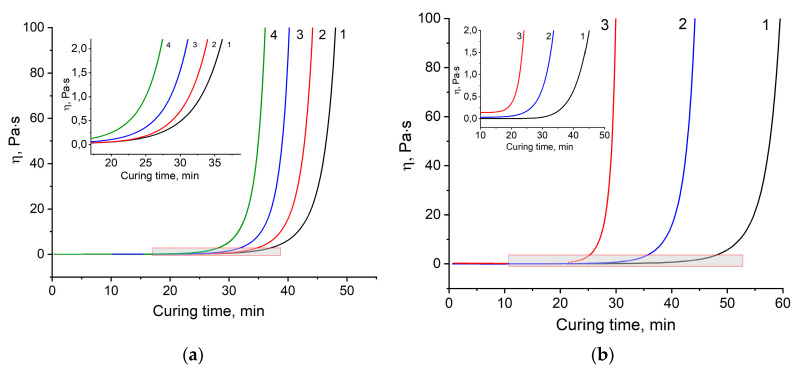
Dependencies of viscosity *η* on curing time: (**a**) at 170 °С for formulations 1–4, (**b**) at 160 °C (1), 170 °C (2), 180 °C (3) for formulation 2.

The process of curing the thermoset system up to the gel point can be studied by rotational viscometry, which allows one to register the change in rheological properties over a sufficiently wide range of viscosities. Viscosity dependences on the curing time at operation: 160, 170, and 180 °C for the four formulations are shown in Figure 2.

Figure 2 shows the rapid growth of viscosity for all compositions, followed by gelation. This dramatic increase is preceded by very low values of viscosity being less than 0.25 Pa·s. This fact limits the usage of this binder for prepreg technology, where minimum viscosity at the curing temperature should be around 1 Pa·s. Thus, we supposed that developing binders could potentially fit vacuum infusion technology, resin transfer molding (RTM), or resin film infusion (RFI).

It was found that for all formulations studied, the change of viscosity η from the curing time τ change could be satisfactorily described by the exponential equation:

η = η_о_ · exp(k_η_ · τ) (1)

where η_0_ is the initial viscosity, Pa∙s; k_η_ is the viscosity increase rate constant, min^−1^.

From Equation (1), represented in semilogarithmic coordinates:

lnη = lnη_о_ + k_η_ · τ (2)

The values of the viscosity increase rate constant were graphically determined (Table 2).

The time dependence of lnη for all systems near the gel point deviates from a linear character in the direction of a faster increase in viscosity. Therefore, when linearizing the dependences to determine the rate constants of viscosity increase, the last sections were not taken into account.

As can be seen from Table 2, the k_η_ constant increases with the modifier content to 20%, which indicates an increase in the gelation rate, but with a further increase in the amount of EP up to 30%, the value of the viscosity increase rate constant does not change.

Equation (1) is true, at least, for values lower than η ≈ 3 × 10^3^ Pa·s. Moreover, it is possible to characterize the “engineering” zone of material processing as the zone that ends when a high viscosity level is reached (e.g., 10^3^ Pa·s). It is close to the engineering limit when the material can still be regarded as a liquid. Hence, this viscosity value practically meets the acceptable technological criteria for gelation.

However, the true gelation time, which corresponds to the condition η→∞, cannot be estimated with Equation (1).

The most reasonable way to determine the gel point using the rotational viscometry method is to determine the maximum possible viscosity of the system and to plot the dependence of the reciprocal viscosity 1/η on time at the final stages of curing [23,24,25]. This dependence at the final stage of the experiment is usually well approximated by a straight line, the intersection of which with the x-axis determines the time when the system reaches an infinite viscosity, i.e., a gel point τ_gel_.

As can be seen from Table 3, the gelation time monotonously decreases both with increasing temperature and with increasing EP content in the system to 30 w%.

The gelation time τ_gel_ and the viscosity increase kinetic rate constant k_η_ in Equation (2) are related by a simple dependence:

(τ_gel_)^−1^ ~ k_η_ (3)

**Figure 3:**
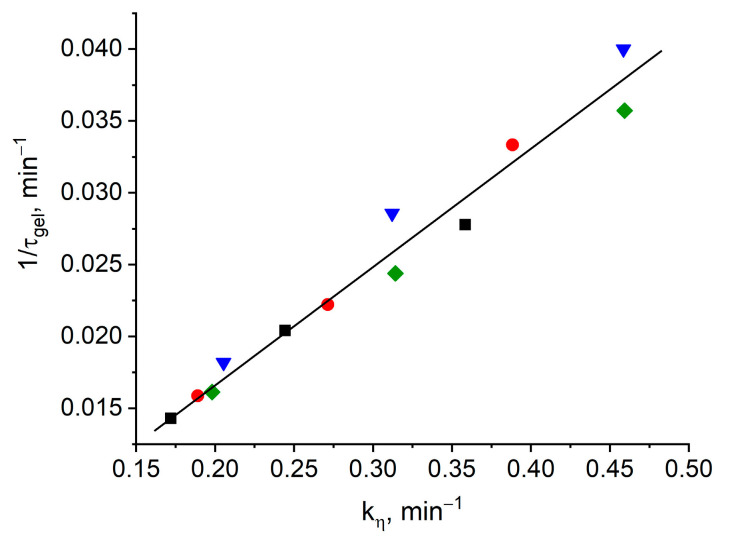
Dependence of the inverse gel time (τ_gel_^−1^) on the viscosity increase rate constant (k_η_) for formulation 1 (square), 2 (circle), 3 (triangle), 4 (rhomb).

Obviously, when this dependence is fulfilled, all points must appear on one straight line, which is actually observed in Figure 3.

The temperature dependence of the “viscometric” curing rate, characterized by the value of the constant k_η_ in Equation (1), can be represented by the Arrhenius Equation:

k_η_ = k_о_ · exp(−E_η_/R·T) (4)

where k_0_ is the preexponential factor, E_η_ is the effective activation energy of the curing process, R is the universal gas constant, T is the temperature (K).

If we present Equation (4) in logarithmic coordinates, this will allow us to estimate the effective activation energy of the curing process E_η_.

Since k_η_ and τ_gel_ depend on temperature, equation (3) can be satisfied only when the values of the activation energy of the process, calculated from the values of k_η_ and τ_gel_, are close to each other.

On average, the activation energy values (Table 4), calculated both by k_η_ and by gelation time, grow with an increase in the content of EP in the mixture up to 20 wt%, a further increase in growth in its amount leads to an insignificant decrease in Е_a_ values.

As can be seen from the obtained data, the absolute discrepancy between the activation energy values calculated from k_η_ and τ_gel_ is less than 5 kJ/mol, which is in extremely satisfactory agreement with the literature data on the variation of the values of the specified parameter, determined by different methods for one system.

It is known that the nature of the increase in viscosity in the process of curing epoxy resins is complex. The increase in viscosity is determined by the change in molecular weight and the structure of the curable oligomer molecules. It is believed that when building the dependence of viscosity on time in logarithmic coordinates, the obtained curves can be divided into two or three linear sections, each of which obeys a power law according to the Malkin–Kulichikhin equation [26]:

η = (f · k · τ)^n^ (5)

where f is the functionality of the oligomer; k is the viscosity increase rate constant (min^−1^); τ is the time (min); n is a constant.

Moreover, each of the linearized regions corresponds to certain structural transformations in the curing system: when n = 1, the molecular weight of the linear chains of the oligomer increases; when 1 < n < 3.5, a fluctuation network of entanglements of macromolecules that have reached a sufficient length to form it arises; at 3.5 < n < 4.5, the three-dimensional cross-linking process begins to predominate until the system loses its fluidity, i.e., until gel point.

Since in this work, the dependences of viscosity on time in logarithmic coordinates could not be approximated by straight lines, in the development of the ideas presented in [27], instead of Equation (5), its modified form (6) was used:

[η(τ)]^1/n^ = f · k · τ (6)

As shown in [28], the behavior of epoxy oligomers with increasing molecular weight, i.e., during the curing process, can be described by the dependency:

(7) $$\eta_{0}(M)\cdot \{\begin{array}{c} aM_{c}^{\alpha }(M<M_{c})\\ bM_{c}^{\beta }\left(M\geq M_{c}\right)\\ cM_{c}^{\gamma }\;\mathrm{for}\;\mathrm{branched}\;\mathrm{polymers} \end{array}$$

where a, b, and c are individual constants of the polymer homologous series (α ≈ 1; β ≈ 3.4–3.5; γ ≈ 4.5), M_c_ is the critical molecular mass at which the nature of the flow of the system changes.

**Figure 4:**
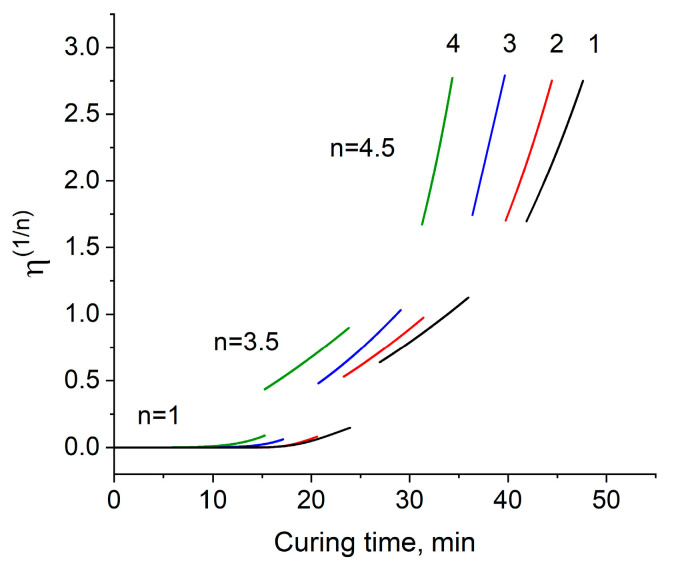
The dependence of viscosity on curing time in the coordinates of the modified Malkin–Kulichikhin equation at a temperature of 170 °C for formulations 1–4.

If we accept the above statements, then we can actually notice a correlation between Equations (6) and (7). Obviously, this theoretical assumption can be easily verified by plotting dependencies in the coordinates η^1/n^ (t) with a subsequent approximation with straight lines in the areas corresponding to the values of n (Figure 4).

As can be seen from Figure 4, the obtained approximation does not quite agree with the assumption made—it turned out to be impossible to completely linearize the last part of the increase in viscosity. It can also be noted that with an increase in the content of the modifier, the duration of all stages of the process is reduced, especially this is clearly seen in the first part of the curve, which determines the growth of molecular weight of the linear chains. Due to 4–6 functional radicals and rigid phosphazene ring of EP, a fluctuation network arises almost 1.6 times as fast for formulation 4 compared to formulation 1 being 15 min and 24 min, respectively. The duration of the second stage of the growth of the fluctuation network and the third stage of the three-dimensional cross-linking process also gets shorter but not so great as the first one.

The obtained values of the characteristic times were compared with the gel formation times for these systems, obtained by the classical approach (Table 5).

All these changes in the rheology of modified compositions curing may be caused by the influence of multifunctionality of phosphazene containing 4–6 reactive epoxy groups, the large molecular mass of the oligomer, and its structure.

The difference in molecular mass, that is, 390 g/mol for ED-20 and 1000–1800 g/mol for EP fraction, results in a difference in viscosity of the neat components, that is, 18.4 Pa·s at 25 °C for ED-20 compared to solid-state at 25 °C for EP fraction (200 Pa·s at 40 °C). This fact influences the viscosity of the compositions till they start to cure; practically, it determines the processing window for binders. The acceleration of viscosity increase during curing is, on the other hand, influenced by high multifunctionality of the modifier that leads to denser network formation, which was proved in our previous physicochemical study [8] by the decrease in the molecular mass of the interjunction segment calculated from the rubbery modulus (determined by dynamic mechanical analysis and by the monotonic increase in the glass transition temperature. The denser the network at any stage of curing, the higher the viscous binder is. Thus, we propose it to be the main reason for the acceleration of viscosity increase in our work.

In addition, it should be mentioned that we propose acceleration of viscosity increase not to be connected to the curing rate. There is no acceleration factors in the structure of epoxyphosphazene resin (Figure 1), moreover epoxy group content in it even lower than in ED-20 being 17−19% and 20−22% respectively.

This work is the first one that revealed study of hot curing process of epoxy-amine binder with epoxyphosphazene represented in Figure 1. However, there are related works [10,29] published by our research group. They were dedicated to the cold curing process of epoxy-amine binder with epoxyphosphazene of that type. In this process the addition of 20wt% the epoxyphosphazene to the binder reduces gelation time by around 1.5 times, while in hot curing process it decreases only by approximately 1.2 times. Cold curing process passes with much more stoichiometric hindrance rather than hot one due to the bulkiness of epoxyphosphazene molecule and the lack of heat for its motion. The study [10] shows that changes of mechanism in cold curing process from second-order reaction to a self-inhibited second-order reaction upon reaching the isothermal glass transition of the system occur quite early at the degree of conversion around 0.6−0.7. The arise of early diffusion control leads to incomplete cure that results in insufficient mechanical characteristics of polymer. Thus, in order to cope with these difficulties hot curing systems were proposed and studied The effectiveness of modifier usage in hot curing systems was proved by the work [8] that represented the improved physical properties of epoxyphosphazene polymers.

## 4. Conclusions

Epoxyphosphazene as a novel modifier for high-performance epoxy-amine binder changes the viscosity of compositions and their rheokinetics of curing. Processes of viscosity increase are accelerated. These are proved by a decrease of gelation time and rate constant growth.

The change in structure formation corresponds to the characteristic time of the beginning of the three-dimensional cross-linking of the composition, calculated from the Malkin–Kulichikhin equation. All these three stages of linear molecular growth, fluctuation network of entanglements formation, and three-dimensional cross-linking process turn to be shorter with the addition of epoxyphosphazene. We propose the main reason for the acceleration of viscosity increase during curing in our work is the multifunctionality of the modifier and bulkiness of its molecule, that causes the faster free-dimensional network formation, in particular, the dramatic decrease of linear molecular growth duration.

## Figures and Tables

**Table 1:** Formulation of the mixtures.

Formulation Number	Component Content, (%)	
	ED-20 ^1^	EP ^2^
1	100	0
2	90	10
3	80	20
4	70	30

^1^ ED-20—Bifunctional bisphenol -A-based epoxy resin of ED-20 brand; ^2^ EP—epoxyphosphazene oligomer.

**Table 2:** Values of viscosity increase rate constants (k_η_) for formulations 1–4 at different curing temperatures.

Formulation Number	k_η_ (min^−1^) at the Temperature of (°C)		
	160	170	180
1	0.172	0.244	0.359
2	0.189	0.271	0.388
3	0.198	0.314	0.459
4	0.205	0.312	0.459

**Table 3:** Values of gel time (**τ_gel_)** for formulations 1−4 at different curing temperatures.

Formulation Number	Values of τ_gel_ (min) at the Temperature of (℃)		
	160	170	180
1	70	49	36
2	63	45	30
3	62	41	28
4	55	35	25

**Table 4:** Values of activation energies (**Е**_η_) for systems 1–4, calculated by the viscosity increase rate constantand by the time of gelation.

Formulation Number	Values of Е_η_ (kJ/mol), Calculated by	
	k_η_	τ_gel_
1	59.8	54.2
2	58.7	60.4
3	68.6	64.8
4	65.5	64.2

**Table 5:** The characteristic times of the process of structuring at different temperatures for the studied formulations, obtained using different approaches in the analysis of viscosimetric data.

Formulation Number	160			170			180		
	τ_n = 3.4_	τ_n = 4.5_	τ_gel_	τ_n = 3.4_	τ_n = 4.5_	τ_gel_	τ_n = 3.4_	τ_n = 4.5_	τ_gel_
1	28	50	70	24	36	49	18	27	36
2	27	43	63	20	31	45	14	20	30
3	26	45	62	20	30	41	13	19	28
4	22	35	55	15	23	35	12	18	25
